# 蛋白降解靶向嵌合体在非小细胞肺癌治疗中的研究进展

**DOI:** 10.3779/j.issn.1009-3419.2022.102.19

**Published:** 2022-07-20

**Authors:** 琳 姜, 敬博 张, 嘉淇 胡, 海翔 齐, 恒 许

**Affiliations:** 100050 北京，中国医学科学院药物研究所 Institute of Materia Medica, Chinese Academy of Medical Sciences, Beijing 100050, China

**Keywords:** 蛋白降解靶向嵌合体, 肺肿瘤, EGFR, ALK, KRAS, Proteolysis targeting chimeria, Lung neoplasms, EGFR, ALK, KRAS

## Abstract

蛋白降解靶向嵌合体（proteolysis targeting chimeria, PROTAC）通过利用泛素蛋白酶体途径实现对靶蛋白降解，颠覆了传统小分子抑制剂的理念。在非小细胞肺癌（non-small cell lung cancer, NSCLC）常见的突变靶点中，PROTAC技术在临床前研究中已经成功实现了鼠类肉瘤病毒癌基因（kirsten rat sarcoma viral oncogene homolog, *KRAS*）、表皮生长因子受体（epidermal growth factor receptor, *EGFR*）和间变性淋巴瘤激酶（anaplastic lymphoma kinase, *ALK*）等蛋白的有效降解。PROTAC药物以其事件驱动的独特优势，有望克服小分子抑制剂产生的获得性耐药的问题，并对难成药靶点展现出良好的治疗潜力，有望成为NSCLC治疗的新策略。

## Abstract (en)

Proteolysis targeting chimeria (PROTAC) degrades target proteins by utilizing the ubiquitin-proteasome pathway, subverting the concept of traditional small molecule inhibitors. Among the common mutation targets of non-small cell lung cancer (NSCLC), PROTAC technology has successfully achieved the effective degradation of kirsten rat sarcoma viral oncogene homolog (*KRAS*), epidermal growth factor receptor (*EGFR*), anaplastic lymphoma kinase (*ALK*) and other proteins in preclinical studies. PROTAC drugs with their unique event-driven advantages, are expected to overcome acquired drug resistance caused by small molecule inhibitors and show good therapeutic potential for undruggable targets, thereby providing a new strategy for the treatment of NSCLC.

肺癌是我国癌症发病率第一的肿瘤，其5年生存率低，有巨大的未被满足的临床需求^[1]^。非小细胞肺癌（non-small cell lung cancer, NSCLC）占肺癌发病总数约85%^[2]^，在一项1, 200例中国NSCLC患者的研究调查中，大约73.9%的患者至少有一个相关基因突变^[3]^。其中鼠类肉瘤病毒癌基因（kirsten rat sarcoma viral oncogene homolog, *KRAS*）、表皮生长因子受体（epidermal growth factor receptor, *EGFR*）和间变性淋巴瘤激酶（anaplastic lymphoma kinase, *ALK*）是临床中常见的几种突变，靶向突变基因的小分子靶向治疗药物已经成为治疗肺癌的有效方法。同时越来越多的新技术也不断涌现，为NSCLC的治疗提供了新思路。

## 1 蛋白降解靶向嵌合体（proteolysis targeting chimeria, PROTAC）技术及其优势

2001年，Crews和Deshaies的团队提出了PROTAC的概念，通过利用泛素蛋白酶体途径实现对靶蛋白降解^[4]^。PROTAC是一个双功能分子，由靶蛋白配体和E3泛素连接酶配体通过连接臂相连^[5]^。PROTAC分子在进入细胞后，其结构中靶向目标蛋白的配体可特异性地与靶蛋白结合，另一端可以募集E3连接酶，形成目标靶蛋白-PROTAC-E3连接酶三元复合物^[6]^。其中E3连接酶可介导泛素结合酶E2对目标靶蛋白泛素化，经过多轮泛素化后就有了多个泛素标签，三元复合物解离后，多聚泛素化的蛋白会被蛋白酶体识别从而有选择性地降解靶蛋白的水平^[7]^。

小分子激酶抑制剂主要通过占据活性结合位点使蛋白功能缺失发挥作用，是占据驱动，易出现耐药性问题^[8]^。PROTAC不是传统的抑制靶标活性，而是利用泛素调节、诱导靶标蛋白降解，因此它是事件驱动，不需要和靶蛋白的活性位点高强度结合，可靶向传统认为不可成药靶点，并有望克服耐药问题；其次，它在靶蛋白被泛素化之后可以释放出来，循环往复结合靶蛋白和E3连接酶，具有催化特性，低剂量就可以发挥作用^[9]^。目前PROTAC处于快速发展阶段，正在成为肿瘤治疗的新策略。

## 2 PROTAC治疗NSCLC的研究进展

### 2.1 靶向降解*EGFR*治疗NSCLC

#### 2.1.1 *EGFR*抑制剂研究进展

*EGFR*在10%-15%的NSCLC患者中存在突变^[10]^。靶向治疗已经成为肺癌患者的标准治疗方法，*EGFR*酪氨酸激酶抑制剂（EGFR-tyrosine kinase inhibitors, EGFR-TKIs）也随之成为药物研发的热点，已上市的*EGFR*-TKIs主要分为三代：第一代EGFR-TKIs如吉非替尼、厄洛替尼等对*EGFR* 19del、*EGFR* L858R突变患者有较好的治疗效果，但是许多患者用药后出现获得性耐药，影响了后续的治疗效果^[11]^；第二代EGFR-TKIs如阿法替尼、达克替尼等，虽然通过引入迈克尔受体加强抑制作用，但是在临床上仍不能克服T790M突变^[11]^；第三代EGFR-TKIs如奥希替尼，不仅在一定程度上可以克服T790M突变耐药^[12]^，对NSCLC脑转移也有显著的治疗效果^[13]^，已成为NSCLC存在*EGFR*突变的患者一线用药。其中第三代EGFR-TKIs莫博替尼（TAK-788）对治疗外显子20插入的*EGFR*罕见突变效果出众^[14]^。值得关注的是，患者在使用第三代EGFR-TKIs后会出现如C797S新的突变，因此需要研发对T790M/C797S等突变均有效果的新一代的靶向EGFR通路的治疗药物。目前国内外许多研究团队也正在利用PROTAC技术开展靶向降解EGFR的研究，希望找到一种可以克服*EGFR*耐药突变的有效策略。

#### 2.1.2 EGFR蛋白降解靶向嵌合体（EGFR-PROTAC）研究进展

2018年，Crews团队^[15]^首次利用PROTAC技术对EGFR受体酪氨酸激酶进行了蛋白降解研究，验证了利用PROTAC技术靶向降解EGFR的可行性。其中一个PROTAC分子在HCC827细胞系上可有效降解外显子19缺失的EGFR，在H3255细胞系上可完全降解L858R突变的*EGFR*。另一个PROTAC分子在H1975细胞系上可降解L858R/T790M突变的*EGFR*。该研究首次实现了PROTAC降解突变*EGFR*。

PROTAC分子由靶蛋白配体、E3泛素连接酶配体和连接臂组成，分子量普遍较大，其口服生物利用度以及体内的有效性是PROTAC药物研究中非常值得关注的问题。2019年，Jin团队^[16]^发现了有较好生物利用度的*EGFR*-PROTAC分子，在小鼠体内药代动力学实验中以50 mg/kg的剂量腹腔注射给药8 h后依然可以保持较高的血药浓度。该PROTAC分子对突变型*EGFR*和野生型*EGFR*有较高的选择性。

2020年，Gray团队^[17]^以第四代变构EGFR抑制剂作为配体，进行了靶向降解EGFR的PROTAC药物研究。其中一个分子可以有效抑制携带三突变*EGFR* L858R/T790M/C797S和*EGFR* L858R/T790M/L718Q的Ba/F3细胞的增殖，其增殖抑制的半数抑制浓度（50% inhibitory concentration, IC_50_）分别为0.041 μmol/L和0.028 μmol/L。同时在细胞水平上发现该PROTAC分子和奥希替尼联合使用时可以增强抑制细胞增殖的能力，说明通过激酶抑制剂和蛋白降解剂的双机制调节可以产生协同效应。

PROTAC分子在靶蛋白泛素化以后，可循环往复地结合发挥作用，具有催化特性，相比于小分子抑制剂有望减少给药次数。在靶向降解*EGFR*的研究中，一些研究团队也证实了PROTAC这一优势。2021年，Jiang团队^[18]^在研究中发现PROTAC分子在细胞水平上停止给药后的72 h内仍然可以持续降解L858R/T790M突变的*EGFR*。Zhang团队^[19]^在2021年的一项研究中也证实了这一现象，在停止给药后48 h内其PROTAC分子可持续降解Del19和L858R/T790M突变的*EGFR*。

值得关注的是美国C4 Therapeutics公司研发的靶向降解*EGFR* L858R的PROTAC分子CFT8919已进入到临床前研究阶段，该化合物口服有效、可以透过血脑屏障、对中枢神经和*EGFR*耐药突变均有较好效果，有望近期进入临床研究^[20]^。

受到小分子双重抑制剂启发，2021年Li团队^[21]^设计合成出可同时靶向降解EGFR和PARP两个蛋白的Dual PROTAC分子，该项研究也为PROTAC技术开拓了新的研究方向。乏氧是许多肿瘤细胞的特征之一，利用肿瘤细胞乏氧的特性开发有针对性的药物，可以提高药物对肿瘤细胞的选择性。2021年，Zhang团队^[22]^将带有乏氧激活的离去基团HALG引入到PROTAC中，设计出的乏氧激活PROTAC分子可在肿瘤组织中被激活而释放出活性降解剂。实验结果显示，在正常氧的环境中，该化合物对HCC4006细胞内的EGFR 19del没有降解作用，而在乏氧条件下可实现对*EGFR* 19del的部分降解，从而提高了药物治疗的精准性。

### 2.2 靶向降解ALK治疗NSCLC

#### 2.2.1 ALK抑制剂研究进展

约4%的NSCLC伴有*ALK*融合基因^[23]^。棘皮动物微管相关蛋白样4（echinoderm microtubule associated protein like 4, *EML4*）-*ALK*融合基因是肺癌中最主要的*ALK*融合基因，有研究^[24]^证实EML4-ALK融合蛋白能够在体内诱导肺癌的发生。目前已有三代ALK抑制剂上市，克唑替尼是首个获得FDA批准上市的ALK抑制剂^[25]^。第二代ALK抑制剂阿来替尼是治疗ALK阳性NSCLC的首选药物，并且改善了克唑替尼治疗脑转移不佳的问题^[26]^。此外，常见的第二代ALK抑制剂还有布加替尼和塞瑞替尼。在临床上第三代ALK抑制剂劳拉替尼已经成为克服*ALK*耐药突变的最后保障^[27]^。当前有研究团队正在利用PROTAC技术开发靶向ALK的降解剂，有望为*ALK*耐药突变的患者寻找一份新的屏障。

#### 2.2.2 ALK蛋白降解靶向嵌合体（ALK-PROTAC）研究进展

2018年，Gray团队^[28]^报道了首个可靶向降解ALK的PROTAC分子，开启了ALK蛋白降解领域的新篇章。实验结果表明，该PROTAC分子在H3122细胞系上对ALK的半数降解浓度（50% degradation concentration, DC_50_）仅为10 nmol/L。进一步研究发现该分子除了降解ALK蛋白，对其他靶点蛋白如PTK2、FER、RPS6KA1和Aurora A也有一定的降解能力，但是对表达有L1196M、C1156Y和G1202R耐药突变的*EML4-ALK*的降解能力还有待提高。选择合适的ALK配体，提高对ALK蛋白的选择性和对耐药突变的降解效果，是未来ALK-PROTAC研究中需要重点关注的方向。

2018年，Jin团队^[29]^对发现的靶向降解ALK的PROTAC分子在小鼠体内进行了药代动力学研究。该PROTAC分子以50 mg/kg的剂量进行腹腔注射给药12 h后仍然有较高的血浆暴露量。2018年，Hwang团队^[30]^报道了可在体内降解ALK的PROTAC分子，该分子在H3122小鼠异体移植瘤模型中通过注射给药的方式显著抑制肿瘤的生长，且对动物体重没有明显的影响。2021年，Jiang团队^[31]^开发了口服体内有效的ALK-PROTAC分子。该化合物在大鼠药代动力学研究中，以10 mg/kg口服给药后，4 h血药浓度达到最高，口服生物利用度为16%。PROTAC分子虽然具有强大的催化特性但也存在潜在的问题，例如除了降解肿瘤中的靶蛋白，也可能会导致正常细胞中的靶蛋白不受控制地降解。光作为一种无创、时空可控的外源刺激，近年来在肿瘤的诊治领域得到广泛的关注^[32]^。2020年，Wei团队^[33]^报道了一种光诱导激活的PROTAC分子。实验结果表明，该分子在体外不经紫外光照射时不能降解ALK蛋白，而经过紫外光照射后光敏基团脱除，则可靶向降解ALK蛋白。

### 2.3 靶向降解KRAS治疗NSCLC

#### 2.3.1 KRAS成为治疗NSCLC的新靶点

*KRAS*在细胞的生存和周期进程等方面发挥重要作用，是NSCLC中最常发生突变的基因之一^[34]^。*KRAS*突变在肺腺癌中的发生概率约为30%，在肺鳞癌中发生的概率约为5%^[35]^。在肺癌中常发生的颠换型突变有G12C、G12V、G12A、G12R，转换型突变有G12D和G12S。*KRAS*突变会引起突变RAS蛋白生成，诱导下游信号转导通路发生激活，导致肿瘤细胞生长、增殖和存活^[36]^。

RAS蛋白表面光滑，缺乏传统的可成药口袋，RAS蛋白与GTP的结合能力远高于ATP，这些都是研发KRAS抑制剂的难点，KRAS也一直以来被认为是不可成药靶点^[37, 38]^。近年来针对*KRAS*突变的小分子抑制剂研发取得突破性进展，美国安进公司的AMG510已被美国食品药品监督管理局（Food and Drug Administration, FDA）批准上市，它通过与突变的半胱氨酸共价结合，来达到抑制KRAS G12C的目的^[39, 40]^。

#### 2.3.2 KRAS蛋白降解靶向嵌合体（*KRAS*-PROTAC）研究进展

开发靶向KRAS的蛋白降解剂可以作为调节突变*KRAS*的补充策略。2020年，Crews团队^[41]^报道了首个可降解*KRAS* G12C的PROTAC分子并在肺癌细胞上进行了深入研究。该化合物在NCI-H2030细胞系上可有效降解*KRAS* G12C。此外，他们还在5种不同的*KRAS* G12C突变细胞系中测试了该化合物的降解能力，DC_50_值均在0.25 μmol/L- 0.76 μmol/L之间。实验结果说明PROTAC分子可以降解突变的*KRAS*，是PROTAC技术靶向不可成药蛋白领域一项重要突破。

## 3 PROTAC技术治疗NSCLC的展望

PROTAC技术现已知可以降解80多个靶点的100多种蛋白，在治疗肿瘤、病毒感染以及神经退行性疾病等领域均有深入的研究。PROTAC技术作为一种新兴的蛋白降解技术，具有靶向不可成药靶点、克服耐药性等小分子抑制剂不可比拟的潜在优势，正处于快速发展时期。同时值得关注的是，相比于小分子药物，PROTAC分子的分子量普遍较大，理化性质以及口服生物利用度等通常不够理想，对肿瘤组织内异常蛋白的特异性降解还有待提高等，这些方面也是PROTAC技术正需克服的一些挑战。由美国Arvinas公司研发的靶向降解雄激素受体PROTAC分子ARV-110和雌激素受体ARV-471已进入临床II期试验阶段，初步证实了PROTAC分子在人体上的基本安全性和有效性，对整个PROTAC药物研发领域的蓬勃发展起到了重要促进作用。目前针对*EGFR*、*ALK*以及*KRAS*等NSCLC治疗中的重要靶点都已研发出具有体内外活性的PROTAC分子，基于这些分子进一步的优化以及系统的临床前评价，相信不久就会有靶向降解上述靶点蛋白的PROTAC药物进入到临床研究阶段，有望为NSCLC患者提供一种全新的治疗手段。
